# Our New York Letter

**Published:** 1887-08

**Authors:** 


### Correspondence.

### OUR NEW YORK LETTER.

EXAMINATION OF CANDIDATES FOR MEDICAL POSITIONS IN NEW YORK HOSPITALS—DR. WOOLSEY JOHNSON—A CASE OF PECULIAR DELIRIUM, ETC.

*Editors Atlanta Medical and Surgical Journal:*

For several years now all candidates for medical positions in any of the city departments of New York have been obliged to pass a civil service examination before receiving their appointments. This might appear like a very radical change of methods for a city where politics is so strong a power, but in certain respects the difference is more apparent than real. When a physician wishes to obtain one of these positions, he is obliged to go through a course of considerable formality before becoming a proper subject for appointment. In the first place he must obtain at the city hall an application blank, which in itself is quite a formidable looking document. It calls for answers of no less than twenty questions, and the candidate must give his name, age, and, among other things, tell if he has ever been arrested, if he uses intoxicants, if he has any defect in sight, hearing, etc. The application must be endorsed by four reputable citizens and then sworn to before a notary public, and the slightest error in filling out this paper is considered sufficient cause for its return to the applicant for corrcetion. After this has been filed the doctor is informed when and where the next examination will take place. At the last civil service examination for Sanitary Inspectors in the Health Department there were over one hundred candidates for five vacancies. The examination was a written one, and the questions were included under ten principal headings, four-and-a-half hours being allowed for answering these questions, which gave each man, and woman also, ample time. Everything pertaining to these examinations seems to be conducted in a perfectly fair manner, and the competitors are known to the examiners by number only, and not by name. It would appear from the papers that the questions given are intended to enable any man possessing a fair medical knowledge to obtain the necessary 70 per cent. and pass, and very few applicants are rejected at this stage of the proceeding. But it must be remembered that passing this examination only makes the candidate *eligible* for appointment, and the list of eligible ones is then sent to those having the power of appointing, from which they select whom they choose. It does not necessarily happen that those passing the best examination will be appointed, for the man who only obtained 70 per cent. is just as liable to obtain a position as the man with 100 per cent. is to be left without one. One notable effect of the civil service is that the number of applicants for appointment is far less than it formerly was, as the idea of having to pass a medical examination frightens off a good many of the timid ones, and some think that the political element is yearly becoming weaker.

Dr. Woolsey Johnson, who has been a health commissioner since 1881, died recently from Bright's disease. He was a graduate of Princeton College and studied medicine at the College of Physicians and Surgeons in this city. Dr. Johnson had been attending physician to the New York Hospital, and was also connected with the Eye and Ear Infirmary, and a member of several prominent medical societies. The vacancy caused by his death will be filled by Mayor Hewitt, who will appoint Dr. Joseph D. Bryant to the position. Dr. Bryant is Professor of Surgery in the Bellevue Hospital Medical College and Surgeon-General on Governor Hill's staff.

At a medical discussion which I listened to a short time ago, a case of rather peculiar delirium, occurring during an attack of rheumatism, was related. The patient was a man aged fifty, a hard drinker, and being attacked with acute articular rheumatism, his physician ordered fifteen grains of salicylate of sodium to be taken every three hours. He had taken four doses during the day, but complaining of considerable pain in the evening, ten minims of Magendie's solution of morphine were administered. Soon after he began to complain of buzzing in the ears and deafness and a mild delirium developed. The salicylate was discontinued for about twelve hours, and on the following morning, as the patient was rational again, it was ordered as before. That night the delirium re-appeared and the salicylate of sodium was again stopped. As he was becoming very excitable, another ten minim injection of Magendie was given with the view of quieting him, but it only seemed to intensify the delirium, for he soon got out of bed and began to walk about the room shouting loudly and acting in the most violent manner. Two doses, each containing ten grains of chloral and thirty grains of bromide of sodium, were then given at an interval of half an hour, but, strange to say, after each dose he became more excited, so no more was given. On the following morning, his condition being unchanged, he was given chloral, gr. xv; ext. hyoscyamus, fl. and ext.; conii fl. aa. qtts. xv. This appeared to be effectual, for he soon became quiet and went to sleep and did not awake for seven hours. On awaking he was still somewhat irrational and continued so for about a week. Although in his own home, he was unable to tell where he was, and imagined there were holes in the wall and wires running across the rooms. The temperature never rose much above 102° F. There was no tremor of the tongue or hands. As the patient had been treated for alcoholism several times with hypodermics of morphine, which always had a soothing effect upon him, his physician did not consider the delirium due to this drug. It did not appear like ordinary alcoholic delirium, for the bromide and chloral mixture seemed to exaggerate it, and it was afterwards learned that the patient's wife had given him a dose of about two ounces of whisky, which produced no quieting influence whatever. The fact that his delirium continued so long after sleep had been induced was another peculiar feature and unlike that ordinarily caused from alcoholism. His physician was inclined to believe that, as the delirium began with buzzing in the ears and other symptoms of salicylic acid poisoning, it was due to the effects of this drug, acting more intensely from personal idiosyncrasy and alcoholic habits of the patient. It was thought that some impurity in the manufacture of the acid might also have been causal, as it had been stated that salicylic acid should always be made from the willow bark, or disagreeable delirium might follow its use.

At this season there is rather a lack of medical news, as many of our prominent lights of the profession are absent from the city; the medical colleges are closed and most of the societies have adjourned for the summer. A few of our well-known doctors have arranged so as to live in the country during the summer, and yet partly attend to their city practice by coming to New York every day, or some only on certain days, holding regular office hours and returning again to their out-of-town homes the same day.

We have just been passing through a very prolonged spell of unusually hot weather, which has had the effect of driving out of the city nearly all who could possibly go. As a result we have also had a sudden increase in infant mortality and many cases of sun-stroke among those exposed to the severe heat. The children of the poor, however, will receive better attention than they did last summer, for a corps of fifty physicians will be appointed in a few days to go from house to house and render gratuitous medical service to these little unfortunates.

New York, July, 1887. A. F.

